# Multisensory Integration and Child Neurodevelopment

**DOI:** 10.3390/brainsci5010032

**Published:** 2015-02-11

**Authors:** Emmanuelle Dionne-Dostie, Natacha Paquette, Maryse Lassonde, Anne Gallagher

**Affiliations:** 1 Sainte-Justine University Hospital Research Center, Montreal H3T1C5, QC, Canada; E-Mails: natacha.paquette@umontreal.ca (N.P.); maryse.lassonde@umontreal.ca (M.L.); 2 Centre de Recherche en Neuropsychologie et Cognition (CERNEC), Departement of Psychology, University of Montreal, C.P. 6128, Montreal H3C3J7, QC, Canada

**Keywords:** infancy, multisensory integration, neurodevelopment, cognition, developmental dyslexia, attention deficit disorder with/without hyperactivity

## Abstract

A considerable number of cognitive processes depend on the integration of multisensory information. The brain integrates this information, providing a complete representation of our surrounding world and giving us the ability to react optimally to the environment. Infancy is a period of great changes in brain structure and function that are reflected by the increase of processing capacities of the developing child. However, it is unclear if the optimal use of multisensory information is present early in childhood or develops only later, with experience. The first part of this review has focused on the typical development of multisensory integration (MSI). We have described the two hypotheses on the developmental process of MSI in neurotypical infants and children, and have introduced MSI and its neuroanatomic correlates. The second section has discussed the neurodevelopmental trajectory of MSI in cognitively-challenged infants and children. A few studies have brought to light various difficulties to integrate sensory information in children with a neurodevelopmental disorder. Consequently, we have exposed certain possible neurophysiological relationships between MSI deficits and neurodevelopmental disorders, especially dyslexia and attention deficit disorder with/without hyperactivity.

## 1. Introduction to Multisensory Information

Most species, including humans, are equipped with various highly specialized sensory systems that give them access to numerous types of information on the surrounding environment. Each sensory modality gives us a unique outlook on the world: Color, for instance, can only be perceived through sight, sound through hearing and temperature feel through the somatosensory system. However, our surroundings never cease to present us with situations that stimulate several senses at once. In day-to-day life, events are rarely unimodal; they are multisensory experiences, deriving from a combination of information acquired through several sensory modalities. The brain integrates this multisensory information to provide a complete and coherent representation of what is being perceived and consequently for appropriate behavioral responses to be generated [1]. For example, if we hear the sound of a siren at a distance and then see a vehicle approaching, the association of visual image and sound leads us instantly to perceive an ambulance. Our perception and behavior are not based on the juxtaposition of independent sensory experience but on the global impression resulting from the combination of information acquired through various modalities. This phenomenon has been called Multisensory Integration (MSI).

In many cases, the survival of an organism depends on appropriate responses to multisensory stimuli. Therefore, our ability to integrate sensory information becomes a fundamental element of our everyday behavior, allowing us to identify events and apply optimal responses to these events. From that perspective, many studies conducted to investigate the behavioral effects of a multisensory presentation have shown that it has beneficial effects. Incidentally, it has been noted that the combination of two sensory modalities could, in various contexts, decrease response time [2,3,4], increase target detection accuracy [5,6], increase acuity in one of the modalities [7], reduce detection thresholds [8] and even facilitate and optimize learning applied to a single sensory modality [9]. Furthermore, these facilitative behavioral benefits are noted regardless of which sensory modalities are being tested.

To improve our understanding of the benefits afforded by the simultaneous presentation, studies have used various type of stimulation. For instance, several studies have shown behaviorally that viewing a stimulated body part can enhance tactile detection and perception at the stimulated site [5] and that this visuo-tactile enhancement may last up to minutes [7,10]. Thus, using visual and proprioceptive stimuli, seeing one’s own arm may shorten the response time to an unseen tactile stimulus to the arm [2]. In return, somatosensory information can also facilitate visual processing [11]. For instance, the response time to a light projected on one’s own finger is shorter than when the light is projected on an object far removed from the body [11]. Similarly, studies have shown that vibrotactile stimuli (for example, short vibrations applied to the palm) shorten the response time to visual stimuli [3]. Finally, the integration of tactile and auditory inputs was also investigated, albeit succinctly, revealing that tactile stimuli can be beneficial to auditory perception. It appears that a tactile signal, even non-pertinent, can sometimes facilitate the detection of a sound, thus increasing the impression of loudness [6,7,8]. In that perspective, research in healthy adults has repeatedly shown behavioral benefit of MSI that might be linked to the underlying ability of the brain to integrate different sensory inputs related to a single event [12].

## 2. MSI and Development

### 2.1. Innate or Acquired

Although MSI capacity has been extensively studied in adults, little has been done to study it in young children. The first important question would be: Does this capacity exist at birth, or does it develop as a result of experience? One school of thought (early integration) argues for the innate nature of MSI while another (late integration) emphasizes the role of experience in the development of MSI.

#### 2.1.1. Early Integration Approach

According to the Early Integration Approach, the nervous system is multisensorial right from its early development stage, possessing the capacities to detect redundant aspects of the surrounding environment [13]. In support of this approach, Bower, Broughton and Moore (1970) [14], have observed that infants are able to move their hands toward visual targets as early as six days after birth, which indicates that hand-eye coordination occurs very early on in life. Even in the first months of life, infants are able to perceive and derive meaning from the abundance of multisensorial information. Although the literature on infants is relatively recent and small, a few authors have suggested that MSI is not a unitary process and that different mechanisms might be implicated depending on the specific type of multisensory interaction [15].

Bahrick and Lickliter (2000) [16] proposed the “intersensory redundancy hypothesis”, in order to explain how infants perceive coherent, unified multimodal objects and events through different sensory modalities. This hypothesis refers to the presentation of the same information spatially coordinated and temporally synchronous across two or more sensory modalities, and is only possible for amodal properties that are not specific to a single sense modality (e.g., shape, rhythm, duration, intensity) [17]. In other words, regardless of which sensory modality, similar qualities are perceived when we integrate information. For instance, when we hear and look at a bouncing ball, we detect that the auditory and visual stimulations that originate from the same location share a common tempo and rhythm. This sensitivity to amodal properties allows the infant to direct his attention to unitary and meaningful events in which information from different sensory modalities originates from a unique point of origin [18,19].

Studies on the cross-modal transfer of information from touch to vision revealed that neonates are able to process and encode shape information about manually experienced objects and to discriminate between subsequently presented visual objects [20,21]. Newborns are also able to visually recognize the texture that they previously felt and tactually recognize the texture that they previously saw [22]. Others have reported that one-month-old infants can benefit from the tactile-oral properties of an object during visual recognition, showing a clear visual preference for objects with which they had been familiarized through oral presentation [23,24,25].

It has been demonstrated that the ability to perceive audio-visual relations also emerges early in human development. For example, Lewkowicz and Turkewitz (1980) [26] have shown that three-week-old infants responded to the equivalence of different levels of auditory loudness and visual brightness inputs on the basis of intensity. Therefore, sound presented concurrently with visual stimulation modified visual behavior. It has been shown that in the first few months of life, infants benefit from temporally and/or spatially associated multisensory information [16,27,28]. While temporal synchrony refers to the capacity to specify whether a particular sound and image go together or not, spatial synchrony concerns whether signals in diverse modalities comes from a common or different location. The importance of synchrony for infant perception has been well documented [29,30,31] and is also reflected in the intermodal preference procedure, in which the bimodal information is typically presented synchronously. It has been reported that on the basis of synchrony, newborn infants are able to associate objects and linguistic stimuli [32] and that just a few hours old infants can learn sight-sound pairings [31]. Bahrick (2001) [28] found that early as four weeks after birth, infants are sensitive and able to learn arbitrary relations between audiovisual inputs, Moreover, it has been demonstrated that infants between three and six months old have the ability to detect the difference and discriminate temporal information of audio-visual inputs [33,34]. In addition, four-month-old infants can connect and bind visual objects to the specific sounds produced by these objects [35,36]. For instance, they are able to look longer at a puppet whose bouncing rate is the same as the rate at which the sound occurred [35]. These signs of integration, detected early on in life, remind us that the temporal synchrony is an important characteristic for MSI.

Behavioral benefits of early integration are imperative in the development of face and emotion recognition and are important features in a child’s abilities to adapt to his environment later in life. Studies using a visual preference paradigm in a multimodal context for human faces have reported interesting results in infants [36,37,38,39]. For instance, two-month-old babies can link phonetic information between voices and lip movements [39] and show an enhanced response when lip movements are synchronized with sounds in contrast to unsynchronized ones [40]. In addition, four-month-old infants can perceive affect (joy, sadness or anger) in words that are supported by audio-visual presentations [41], and discriminate these affects in a multimodal context (*i.e*., establishing relations between faces (visual modality) and voices (auditory modality) [41,42]. Overall, this data might support a form of multisensory association present early on in life, favoring the innate hypothesis for MSI development. However, all studies include post-natal investigations, which limits the opportunity to draw firm conclusions. Studies on children born prematurely could be used to make more compelling arguments in favor of this hypothesis and could be an interesting avenue of research. Also, evidence of multisensory processes in newborns and infants has been demonstrated largely by behavioral data and needs further investigation. Few researches have been carried to study the presence of multisensory mechanisms using neuroimaging techniques. Ethical and practical limitations such as recruitment may have diminished the possibility to undertake such studies on newborns. Nevertheless, the few studies found in the literature will be discussed in Section 2.2.

#### 2.1.2. The Late Integration Approach

In opposition to the Early Integration Approach, the Late Integration Approach emphasizes the acquired nature of MSI. According to this hypothesis, all sensory systems work independently of each other at birth. At this time, these systems are not mature and become increasingly refined during the child’s development. This is a long-term process during which cognitive changes and neuronal reorganization keep going on until adolescence in which the brain must continuously adapt its neuronal networks between sensory and motor communications [43]. In support of this, Putzar and colleagues (2007) [44] have recently shown that temporary visual deprivation in the first two years of life affects the level of audio-visual integration that can be achieved once normal vision is restored. These difficulties also persisted into adulthood [44]. This suggests that a critical period in infant’s development might underlie the emergence and maturation of MSI, therefore reaching maturity ontogenetically late. During the first months of life, contact with multisensory information seems to be a prerequisite to develop and refine integration of these various sources of information. Thus, if relevant experience is not gained in infancy, individuals cannot compensate for this loss later in life [45].

Furthermore, the various senses do not develop at the same rate. For instance, in humans, all the sensory systems are functional at varying degrees by the end of gestation as these systems progress towards full structural and functional maturity. Sensory structures underlying touch seem to be the first to emerge during ontogenesis [46], followed by the onset of functional hearing (third trimester) and the development of the visual system (largely after birth) [47,48]. While these findings indicate that there is a difference in the rate at which the various sensory systems develop between one another, there is also a difference in the way diverse characteristics develop within each of these systems. For instance, visual acuity and contrast sensitivity keep improving until the child is five- or six-years-old [49], while the period between two and five years of age is the time of development of perceptual language [50], which corresponds to the processing of speech signal, both acoustically and visually. A study by Morrongiello *et al.* (1994) [51] found an age effect in their study on object exploration and object recognition in children between 3 and 8 years of age with common objects of different sizes. Their results showed a distinct developmental pattern: Older children were faster, recognized more objects and were more thorough in their exploratory strategies than the younger children [51]. Moreover, object handling skills improve until the child is 8- to 14-year-old [52]. Thus, the child will have to learn to integrate multisensory information during his development [13].

Recent studies conducted on very young infants have also suggested that post-natal experience might contribute to MSI development [53]. Neil and colleagues (2006) [54] have studied the development of eye and head movements in 1- to 10-month-old infants while they were subjected to auditory, visual and audio-visual stimuli. Results showed that only 8- to 10-month-old infants responded significantly faster in bimodal conditions than in unimodal conditions, suggesting that audio-visual integration emerges at a late stage in the first year of life. Other studies have supported the assumption that the integration of different modalities does not become optimal until relatively late in childhood. In contrast to adults, when confronted to different sources of information from various sensory modalities, children do not optimally integrate the information from the two sensory modalities but make their perceptual judgments based only on one or the other sense. This unisensory dominance has been found in visuo-tactile integration task [55], visual and tactile cues for size and orientation discrimination [27], nonvisual self-motion and visual landmark information [56], in judgments of surface slant based on stereoscopic and texture information [57] and in audio and visual space and time perception [58]. According to Burr and Gorri (2011) [59], this unisensory dominance in which the most robust modality is employed to tune the others could reflect a process of cross-sensory calibration. As stated in the previous studies, MSI has not reached maturity in children younger than eight years old [27,55,56,57,58].

A number of authors have also put forward the presence of a MSI temporal window over which the strength of multisensory interactions is dependent on the spatial and temporal synchrony between different sensory inputs [60,61,62]. The more remote in space and time two sensory stimuli are, the less likely they are to fuse. A behavioral study by Hillock and colleagues (2011) [63], reported that the audio-visual integration temporal window is still immature in 10- and 11-year-old children, which supports the hypothesis that the underlying plasticity and maturation of MSI continues through development.

Unlike adults, behavioral studies have reported immature multisensory processing capacities in children and adolescents in audio-visual discrimination tasks [63,64,65] and in other sensory modalities [56]. For instance, Barutchu and colleagues [64,65] reported that multisensory facilitation is still immature in 10-year-old children during a simple audio-visual detection task. In accordance with the Late Integration Theory, results from these studies suggest that information acquired through various sensory modalities might not be integrated optimally in very young infants and that optimal MSI only occurs in children older than eight years [27,56] with changes occurring over a prolonged time course that may extend into adolescence.

Animal studies have corroborated the notion that the brain is immature and must learn to combine the various types of sensory information. One of the most studied neural structure in MSI is the cat superior colliculus (SC), a midbrain structure in which neurons located in the deep layers are responsible to converge multisensory inputs [66]. These multisensory neurons are responsible, among other things, to detect and orient one’s behavior to external events and sensory stimuli [67,68,69,70]. Visual, auditory and somatosensory inputs stimulate the SC and each sensory modality is represented in a map-like representation in which all the different maps overlap each other and are in close topographic register. Therefore, the alignment among the sensory maps is fundamental for multisensory neurons to integrate the inputs from various senses manifesting itself into an adequately behavioral response [71]. Nevertheless, according to Wallace and colleagues (1997, 2006) [72,73] multisensory neurons found in the SC are not present at the cortical and sub-cortical levels immediately after birth and it is only after several months of life and exposure to a multisensory environment that the integration-specialized neurons progressively appear and mature in the cat. Although the appearance of the first multisensory neurons in kittens is at about ten days of age, their ability to integrate inputs from multiple sensory modalities that can be considered adult-like is not seen until three post-natal months [72]. As in the cat multisensory SC neurons, the newborn rhesus monkey also fails to integrate coincident cross-modal inputs [74,75]. Thus, this capacity might indeed be strongly dependent on experiences [73,76].

Early post-natal experience is critical and deprivation of sensory information during this time can lead to an inability to integrate signals neurologically [45,77]. Studies conducted on the effects of sensory deprivation have demonstrated the significance of sensory organ stimulation to the proper functioning and development of sensorineural structures. It is possible that MSI capacities increase in precision over the course of human development and that they are progressively enriched through both the maturation of brain systems and the accumulation of experience. Since newborns from all species are first introduced with a complex and multisensorial environment, they ought to possess some neural mechanisms allowing them to adapt to that environment promptly. Although these coping mechanisms remain fragile and rudimentary, it is these mechanisms that allow their survival. This review exposes a discrepancy in the data revealing that newborn animals cannot perform MSI at birth while there is evidence that newborn humans can. This divergence may be accountable in the fact that animal adaptation is specific to natural environments, and “does not occur when the animal is viewing artificial stimuli such as gratings” [78]. Nevertheless, the question whether MSI is innate or a product of our environment remains debatable and requires further investigation.

### 2.2. Neuroanatomical Correlated of MSI and Pediatric Brain Activity

The recent explosion of EEG’s and neuroimaging techniques has allowed the identification of numerous multisensory convergence zones in the brain. Numerous cortical and subcortical brain regions have been identified to receive afferent inputs from multiple senses. Subcortically, the SC and the basal ganglia as well as association and other cortical regions including the superior temporal sulcus, the parietal, premotor and prefrontal cortex [79,80,81,82] are also implicated in MSI, including feedback as well as feedforward anatomical projections [83,84]. Moreover, recent evidence at the earliest stages of perceptual processing reveals multisensory modulations, activations and connectivity in sensory sensory-specific brain areas [85,86].

To date, knowledge of the development of the neurophysiological processes that underlie MSI comes largely from animal and adult studies. To our knowledge, one recent study using electrophysiology (ERP) [87] has investigated the development of MSI capacities in infants. In this study, Reynolds and colleagues (2014) [87] have shown evidence of enhance neural responsiveness to synchronous audiovisual stimulation compared to asynchronous stimulation in five-month-old infants. Besides, only a few studies have dealt with the underlying cerebral mechanisms of MSI in older children and teenagers. It is in particular the case of Brandwein, Foxe, Russo, Altschuler, Gomes and Molholm (2011) [88] who used ERP to characterize the developmental trajectory of brain processes underlying audiovisual MSI in forty-nine neurotypical children and adolescents aged between 7- and 16-years-old. The data suggest that mature levels of multisensory facilitation are only reached by approximately 15 years of age. Another ERP study in typically developing children aged between 6- and 13-years-old suggests that like adults, they integrate multisensory audio-somatosensory input during multiple stages of sensory information processing [89]. However, a study in magnetoencephalography revealed a robust audio-somatosensory integration response in 11- and 13-month-old infants, which suggest the development of MSI during the first year of life [90].

As outlined above, these studies provide encouraging evidence for cortical MSI in typical children. However, considering the important discrepancy in neurophysiological data, further exploration of MSI in neurotypical children is required. The use of neuroimaging approach is of great interest for the scientific community and could fill the current gap of knowledge on developmental MSI.

## 3. MSI and Cognitive Development

As previously stated, multisensory stimuli provide an enhanced representation of the environment [91] and MSI processes facilitate the speed and accuracy of a variety of behaviors and perceptual processes. The benefits of multisensory information derive from empirical support with infants in various domains showing that multisensory stimulation can enhance early perceptual, affective, and cognitive discrimination. For instance, the combined use of information from several senses has been found to enhance time discrimination abilities in infants [16,33] as well as affect discrimination [41] and even numerical cognition in infants [92,93] and preschool children [94]. Multisensory stimulation promotes heightened attention, perceptual processing and memory in adults as well as in infants [16,95]. Some authors have put forward that the capacity to perceive a complete and coherent representation of multimodal information provides the groundwork for the development of perception, cognition and behavioral abilities, which, in turn, are critical elements to the development of higher-level perceptual and cognitive functions [96,97]. The acquisition of MSI capacity would influence perceptual learning [16] and the acquisition of global cognitive and intellectual abilities [98,99]. Therefore, these MSI capacities in infancy may provide an important developmental foundation for the emergence of cognitive abilities in adulthood.

The premise that MSI can contribute to cognitive development has been supported by studies showing lower intellectual functioning in children with MSI deficits [99,100,101]. Some studies showed that information transfer across different modalities predict verbal performance in school age children, suggesting that primary MSI skills can impact the acquisition of verbal aptitudes [99,100]. For instance, recent findings showed that children who did not demonstrate good MSI skills had weaker verbal processing scores than what was expected when considering their age and global intellectual quotient [101]. Moreover, sensory integration deficits might result in various difficulties such as learning new skills, getting organized, regulating attention and engaging in positive social experiences in some children [102]. These kinds of difficulties in integrating multisensory information might be associated with particular neurodevelopmental disorders in children.

## 4. MSI in Neurodevelopmental Disorders

According to the World Health Organization, neurodevelopmental disorders affect one in six children in industrialized countries [103]. Impacting cerebral growth and development, neurodevelopmental disorders encompass a wide range of disorders, such as intellectual disability, Tourette syndrome, autism spectrum disorder, learning disabilities and attention deficit disorder with or without hyperactivity (ADD/ADHD). Children with neurodevelopmental disorders also represent a significant proportion of students who are struggling or failing in school [104,105]. A better understanding of these disorders thus appears to be a crucial concern, particularly when embracing a therapeutic and preventive perspective. Therefore, the following sections will examine the relationship between MSI deficits and two neurodevelopmental disorders commonly found in children: Developmental dyslexia [106] and attention deficit disorder with or without hyperactivity (ADD/ADHD).

### 4.1. MSI and Dyslexia

Developmental dyslexia is a specific reading and spelling deficit affecting 4% to 10% of the population [107]. According to the Fifth Edition of the Diagnostic and Statistical Manual of Mental Disorders (DSM-V) [106], this neurodevelopmental disorder manifests itself during the years of formal schooling and is characterized by persistent and impairing difficulties with learning foundational academic skills in reading, such as reading fluently with an accurate comprehension, despite average or above-average intelligence [106,108].

Deficits in the phonological domain have consistently been found to be the primary cause of developmental dyslexia [109,110,111,112], although other cognitive deficits, such as in working memory [113,114], executive functions [115,116,117,118], processing speed [119,120,121] and attention [122] have been linked to this disorder. Although a number of conceptual frameworks have been put forward to explain the spectrum of neurological deficits seen in dyslexia, we will review the phonological-deficit hypothesis and the temporal processing hypothesis, two models of particular interest for developmental MSI.

#### 4.1.1. MSI and the Phonological-Deficit and Temporal Processing Hypothesis

The phonological-deficit hypothesis supposes that reading and spelling difficulties result from impaired phonological processes specific to language [107,123,124]. MSI is undoubtedly an important competence in the acquisition of reading skills, since they rely upon rapid and accurate associations between the visual (written) and auditory (verbal) labels [125,126,127]. Regardless of phonological awareness difficulties, authors have suggested that children with dyslexia have difficulties associating verbal labels to the appropriate visual stimuli and thus, establishing appropriate associations between a word and its spelling [127,128]. A number of studies have put forward that unstable letter-speech sound associations could be a critical factor in dyslexia [129,130]. It has been found that children with developmental dyslexia showed significant deficits in processing letter and digit strings (verbal material) [131]. These results receive strong support from a neuroimaging study by Blau and colleagues (2010) [132] showing fundamental deficits in letter-sound integration in children with dyslexia. Similarly, recent findings on the influence of processing speed on reading acquisition in six-year-old children revealed that, whatever the modality (audio-verbal, visuo-verbal, visual, and visuo-visual), children with reading difficulties displayed poor performances in rapid intermodal processing speed in audio-verbal (phonological awareness) and visuo-verbal (rapid naming) tasks [133]. These results support the influence of MSI in rapid naming and phonological processes involved in reading, suggesting that the neural mechanisms underlying audio-visual integration in children with dyslexia differ from normal reading children.

Other studies postulated that not only unimodal [125,134,135] but also multisensory verbal and nonverbal information processing is temporally impaired in children with dyslexia. Some evidence suggests that the core deficit of dyslexia is based on atypical temporal processing of audio-visual multisensory information [125,135,136,137,138]. An interesting paradigm to investigate intermodal temporal processing is the McGurk effect [139]. This effect occurs when an individual sees and hears a speaker producing speech segments that are incongruent with their visual perception. For instance, a classic McGurk effect is hearing the syllable /ta/ while the speaker uttered /ga/. Thus, the auditor usually perceives the sound /pa/. This robust effect in neurotypical individuals was found to be impaired in subjects with dyslexia, suggesting a deficit of MSI. For that matter, instead of perceiving the normal illusion, individuals with dyslexia tend to pronounce the sound perceived visually [140]. Similarly, children with reading difficulties showed low performances in comparing two patterns of brief nonverbal audio-visual stimuli [141]. Moreover, decreased segregation acuity and prolonged cross-modal segregation times were also evident using trains of brief stimuli in three different sensory modalities (audio-visual, audio-tactile and visuo-tactile) in 8- to 12-year-old children with dyslexia, especially in the audio-visual condition [142]. These findings suggest the implication of a general impairment of the nervous system in children with dyslexia when processing audio-visual information rather than a sole deficit in the sensory, motor or phonological systems.

#### 4.1.2. Other Sensory Deficits in Dyslexia

An array of subtle sensory defects unrelated to audio-visual stimuli has also been reported in individuals with dyslexia. For instance, previous studies involving a dual task reported that postural control was highly impaired in children with dyslexia when compared to age- and gender-matched controls [143,144]. Recent findings have also shown that children with dyslexia had difficulty maintaining balance while fixing a point in front of them [145] and that coupling between visual information to body sway was weaker and more variable compare to normal reading children [146]. Poor postural control in children with dyslexia might reflect an inability to integrate multiple sensorimotor inputs, such as proprioceptive signals, that are necessary for proper motor activity. Even though sensory motor deficits were put forth to explain dyslexia, there is a current gap in the literature concerning this hypothesis. Future research on this matter should be considered and would be an interesting avenue to pursue.

Based upon these results, the impairments suggested to underlie the causes of dyslexia could be directly related to atypical audio-visual temporal processing, rapid and accurate associations of visual and auditory stimuli and sensory-motor integration. These findings provide additional information about the neurophysiological causes and evidence of the MSI hypothesis to explain developmental dyslexia.

#### 4.1.3. Anatomical and Structural Differences in Dyslexia and MSI

Recent neuroimaging studies have investigated neuronal activity in multisensory cortical regions of normal readers and individuals with specific learning disabilities in reading. Given its role in language processing, the most consistent finding is altered morphology in the left temporal lobe. For instance, Welcome and colleagues (2011) [147] recently reported reduced asymmetry in gray matter thickness within the temporo-parietal region and smaller brain sizes in the right inferior frontal region in children with reading difficulties. Using fMRI during a phonological task, Temple (2001) [148], reported reduced left temporo-parietal activity in 8- to 12-year-old children with dyslexia compared to normal reading children. Likewise, two magnetoencephalography (MEG) studies from Simos *et al.* (2000) [149,150] supported these results and found decreased left temporo-parietal activity in children with dyslexia while they were reading words and pseudowords. The temporo-parietal region is known to be involved in the integration of letters and speech sounds [151,152], a crucial ability for reading in beginning readers. In addition to atypical activity within the temporal lobe, pediatric functional magnetic resonance imaging (fMRI) studies have also reported deviant patterns of activation [153,154,155] and morphological brain alterations [156] in frontal brain regions of children with dyslexia. One possible explanation for these cerebral differences might be the reduced specialization for processing letters and letter-sound associations.

Altered connectivity patterns in primary sensory and multisensory processing regions were also found in individuals with dyslexia [157,158]. More precisely, the heteromodal superior temporal cortical regions as well as the auditory cortex (Heschl sulcus and planum temporale) have been identified as integration sites for letters and speech sounds in normal readers [151,159]. However, it has been found that children with dyslexia that failed to recognize speech sounds presented together with visual letters (congruent or incongruent) also showed reduced neural integration in the left planum temporale and Heschl sulcus and the left superior temporal sulcus [132]. Overall, these studies suggest that an interrelated network of visual, auditory and heteromodal brain areas might contribute to the skilled use of letter-speech sound integration necessary for learning to read. Reduced congruency between letters and speech sounds in children with dyslexia are likely to indicate less successful letter-speech sound integration [132]. The presence of deviant patterns of activation and morphological brain alterations associated with MSI may play a critical role in the neural basis of dyslexia. Despite the gap of knowledge on this matter, these results highlight the importance of pursuing such neuroimaging studies in order to better understand the impact of MSI deficits on children with reading disabilities.

Atypical development of MSI capacities during infancy can have a momentous impact on children’s neurodevelopment. The previous findings suggest that children with dyslexia have difficulties in MSI and may suffer from integrating sensory cues coming from multiple sources. The hypothesis that MSI deficits might be responsible for impaired reading is still premature. The impact of such difficulties remains unclear and requires greater attention from researchers. However, the development of this integrative ability may offer a cue that may be useful in identifying children likely to develop later difficulties. Therefore, we can speculate that children with dyslexia would take advantage of intervention protocols directed to improve sensory integration.

### 4.2. MSI and Attention Deficit Disorder with or without Hyperactivity (ADD/ADHD)

ADHD occurs in most cultures in about 3% to 5% of children [107,160] and accounts for approximately half of all pediatric referrals to mental health services in the United States [161,162]. ADD/ADHD is a neurodevelopmental disorder characterized by impairing levels of inattention, disorganization, and/or hyperactivity-impulsivity that interferes with functioning or development. Inattention and disorganization entail inability to stay on task, to give close attention to details, to listen when spoken to, to follow through on instructions, to be easily distracted by extraneous stimuli and to have difficulty organizing tasks and activities, at levels that are inconsistent with age or developmental level [107]. Hyperactivity refers to excessive motor activity when it is not appropriate or excessive fidgeting or tapping hands or feet when seated, difficulty to remain seated in situations where it is expected, overactivity, talking excessively, at levels that are inconsistent with age or developmental level [107]. Impulsivity implies a desire for immediate rewards or an inability to delay gratification which can manifest into behaviors such as a difficulty to wait for her/his turn, interrupting or intruding into other people’s activities and making important decisions without consideration of long-term consequences, at levels that are inconsistent with age or developmental level [107]. According to the DSM-V, ADHD symptoms must be present in at least two settings, impact directly on social and academic activities and must be present before the age of 12-years-old [107]. While the predominantly inattentive type (ADHD-I) is the most common subtype in the population (38%–57% of all individuals with ADHD), individuals with the combined inattention-hyperactivity type (ADHD-C) (22%–26%) are more likely to be referred for clinical services [163].

Additionally to attention problems, ADHD is often accompanied by deficits other than those subsumed under the ADHD diagnosis. In terms of cognitive profile, children with ADHD often have difficulty with executive functions (e.g., planning, set shifting, organization, inhibition and regulation of behavior) as well as processing speed and working memory [164,165,166,167,168,169,170,171,172,173,174,175,176]. A high percentage of children with attention disorders also have sensory processing problems, exemplified by behavioral evidence of difficulty modulating sensory responses [177,178,179]. It has been reported that boys with ADHD aged between 6- and 10-year-old have more sensory processing difficulties than neurotypical boys [180]. It has also been suggested that these children may not be perceiving and processing sensory information properly as well as having difficulty producing appropriate responses at school, at home and in the community [181]. Compared with children without neurodevelopmental disorders, children with ADHD exhibit greater difficulties in the sensorimotor domain such as the vestibular and balance control systems [182,183]. For instance, in contrast to neurotypical children, Hassan and Azzam (2012) [184] showed that children with ADHD-C aged between 8- and 10-year-old had lower somatosensory, visual and vestibular ratios by 1%, 9%, and 18%, respectively. According to Guskiewicz and Perrin (1996) [185], this could be the result of a lack of adequate interaction among the three sensory inputs that provide orientation information to the postural control system. Furthermore, children with ADHD also have more difficulties to process tactile [186], visual [187] and auditory stimuli [188,189,190,191]. More precisely, while Hern and Hynd (1992) [186] found that 6- and 12-year-old children with ADHD-C exhibit more soft signs than the normal group on a prototype sensorimotor soft sign battery. Ghanizadeh (2010) [187] demonstrated that children with ADHD give poorer performances on visual acuity and visual field. In the audition realm, a number of studies reported auditory processing problems in children with ADHD [188,189,190,191].

### 4.3. Neuroanatomical Model for ADHD

Studies investigating the neural basis of ADHD have steadily showed structural and/or functional anomalies in cortical areas, basal ganglia, and cerebellar brain regions in children with ADHD [192,193,194]. This circuit has been extensively investigated using methods such as structural volumetric brain imaging and fMRI and has been linked to ADHD behavioral deficits such as inhibitory response, working memory and executive functioning impairments [194,195,196,197,198,199,200,201,202,203]. For instance, using volumetric brain imaging to examine anatomic brain abnormalities, an extensive study compared regional brain volumes in 152 children and adolescents with ADHD aged 5 to 18 years old and 139 age- and sex-matched controls [204]. Children with ADHD exhibited smaller total cerebral and cerebellar volumes in contrast to control children and these volumetric differences persisted into adulthood. Furthermore, frontal and temporal gray matter, caudate nucleus and cerebellar volumes correlated with the severity of ADHD symptoms [204]. Other studies investigating volumetric differences between children with ADHD and typically developing children also reported smaller cerebral volume in frontal and prefrontal cortices [205,206,207]. Such cortical volume reductions were also associated with altered brain activation in sensory cortices (auditory, visual and somatosensory cortices) [184,208], which could explain sensorimotor deficits in this population. The frontal lobes both receive a multitude of inputs from sensory association areas and have influence over a wide region of the nervous system to direct behavior. Moreover, working memory, planning, and reasoning, often affected in ADHD, are associated to the frontal lobes and depend on the recognition and integration of a vast network of signals.

Research evidence in favor of the existence of bidirectional influences between attention and MSI is thus considerable and allows the possibility to make neuroanatomical connections between ADHD and MSI networks. Attentional processes and MSI share subcortical networks such as the SC [209] and operate with cortical regions, including the fronto-parietal and temporo-parietal networks [210]. Moreover, top-down attention is controlled by a fronto-parietal network of brain areas, which sends signals that modulate the sensitivity of neurons in sensory brain regions [211]. Recently, Koziol *et al*. (2011,2012) [192,212] integrated SPD/SMD symptoms in relation to their impacts upon the development of inhibitory control, working memory, academic skill, and behavioral automation in children with ADHD, proposing the first neuroanatomical conceptualization model of sensory processing deficits in ADHD. They suggested that an integrative network of specific brain regions is involved in both cognitive development and sensorimotor integration of the environment [212,213]. According to their model, the cortex encodes specific sensory information (visual, auditory, spatial, perceptual and sensory) by interacting with the basal ganglia that act as a selection mechanism and is important to voluntary motor movement, perceptual learning and inhibitory response. Then, the cerebellum receives sensory information inputs from the involved cortical regions and basal ganglia in order to integrate stimuli and modulate or regulate the intensity of experienced stimulation [212,213]. As previously mentioned, similarly to the neurons in the SC and related structures, previous studies have demonstrated the presence of multisensory neurons in the basal ganglia [85]. Neurons sensitive to visual, auditory or somatosensory modalities have been found in both the substantia nigra (SN) and the caudate nucleus (CN) in which a high proportions of neurons with multisensory properties have been described in these neuronal populations [214,215]. MSI deficits in the CN and the SN may lead to a disruption in the processing of complex sensory stimuli which, indirectly will affect the sensory feedback of motor actions controlled by the basal ganglia. Studies have documented anatomical abnormalities, such as volume decrease, asymmetric and connectivity differences, in the basal ganglia [203,208] and cerebellum of children with ADHD [203,216,217,218,219].

Overall, the neural basis of ADHD has been widely documented by functional and structural studies. Anatomical evidence has provided a reasonable framework for the suggestion that ineffective MSI cortical maps underlying sensory-related behaviors as well as physiological reactivity to sensory stimuli found in children with both ADHD and sensory processing problems may possibly explain their comorbidity.

## 5. Conclusions

One of the most impressive features of the central nervous system is its ability to process information from a variety of stimuli to produce an integrated, comprehensive representation of the external world. Research in neurotypical adults has repeatedly shown behavioral benefits of MSI that might be linked to the underlying ability of the brain to integrate different sensory inputs related to a single event [11]. The benefits of the combined use of information from several senses have been exposed in numerous studies and reveal notably that MSI aids detection and speed response [2,3,4,5,6,7,8,9]. Although MSI capacity has been extensively studied in adults, the literature is scarcer regarding the ability to integrate multisensory information in infants. This review firstly aimed to describe MSI developmental processes of neurotypical infants and children and its neuroanatomic correlates. Moreover, we addressed the question whether the capacity to integrate multisensory information exist from birth or develops as a result of experience. The debate between MSI being innate or acquired remains. Neurophysiological data on animals [59,60,63] and ERP studies on children and adolescents [65,66] suggest that our optimal capacity to integrate multisensory information reaches its maturity late during childhood and is strongly dependent on early experience.

The second section of this article reviewed the presence of sensory integration impairments in children affected with a neurodevelopmental disorder. We first studied dyslexia and paid a particular attention to the phonological-deficit and the temporal processing hypothesis, two models of special interest for developmental MSI. Overall, the literature suggests that an interrelated network of visual, auditory and heteromodal brain areas contributing to the skilled use of letter-speech sound integration might be impaired in children with dyslexia. Studies on ADHD also reported potential alterations of MSI. Children with ADHD often show sensory deficits including sensorimotor [166,167], somatosensory [168], visual [171] and auditory [172,173,174,175] processing impairments. Moreover, anatomical evidence suggested that ineffective MSI cortical maps underlying sensory-related behaviors as well as physiological reactivity to sensory stimuli found in both children with ADHD and sensory processing problems may possibly explain their comorbidity. Although certain neurodevelopmental disorders can cause MSI impairments, research and clinical applications in development are in progress and constitute promising research avenues.
